# Key Signature Genes of Early Terminal Granulocytic Differentiation Distinguish Sepsis From Systemic Inflammatory Response Syndrome on Intensive Care Unit Admission

**DOI:** 10.3389/fimmu.2022.864835

**Published:** 2022-06-30

**Authors:** Sonia Y. Velásquez, Anna Coulibaly, Carsten Sticht, Jutta Schulte, Bianka Hahn, Timo Sturm, Roman Schefzik, Manfred Thiel, Holger A. Lindner

**Affiliations:** ^1^ Department of Anesthesiology and Surgical Intensive Care Medicine, Medical Faculty Mannheim, Heidelberg University, Mannheim, Germany; ^2^ Next Generation Sequencing Core Facility, Medical Faculty Mannheim, Heidelberg University, Mannheim, Germany; ^3^ Mannheim Institute of Innate Immunoscience (MI3), Medical Faculty Mannheim, Heidelberg University, Mannheim, Germany

**Keywords:** gene classifier, granulocytes, infections, sepsis, systemic inflammation, transcriptome

## Abstract

Infection can induce granulopoiesis. This process potentially contributes to blood gene classifiers of sepsis in systemic inflammatory response syndrome (SIRS) patients. This study aimed to identify signature genes of blood granulocytes from patients with sepsis and SIRS on intensive care unit (ICU) admission. CD15^+^ cells encompassing all stages of terminal granulocytic differentiation were analyzed. CD15 transcriptomes from patients with sepsis and SIRS on ICU admission and presurgical controls (discovery cohort) were subjected to differential gene expression and pathway enrichment analyses. Differential gene expression was validated by bead array in independent sepsis and SIRS patients (validation cohort). Blood counts of granulocyte precursors were determined by flow cytometry in an extension of the validation cohort. Despite similar transcriptional CD15 responses in sepsis and SIRS, enrichment of canonical pathways known to decline at the metamyelocyte stage (mitochondrial, lysosome, cell cycle, and proteasome) was associated with sepsis but not SIRS. Twelve of 30 validated genes, from 100 selected for changes in response to sepsis rather than SIRS, were endo-lysosomal. Revisiting the discovery transcriptomes revealed an elevated expression of promyelocyte-restricted azurophilic granule genes in sepsis and myelocyte-restricted specific granule genes in sepsis followed by SIRS. Blood counts of promyelocytes and myelocytes were higher in sepsis than in SIRS. Sepsis-induced granulopoiesis and signature genes of early terminal granulocytic differentiation thus provide a rationale for classifiers of sepsis in patients with SIRS on ICU admission. Yet, the distinction of this process from noninfectious tissue injury-induced granulopoiesis remains to be investigated.

## Introduction

Sepsis is a leading cause of death globally (1), and timely administration of antibiotics is life-saving (2). Given the growing issue of bacterial multidrug resistance, it requires a rational basis (3). There is, however, no gold-standard diagnostic test for sepsis, and routine microbiology ascertainment takes hours to days (4). Consensus definitions have conceptualized sepsis as a systemic inflammatory response syndrome (SIRS) due to infection (sepsis-1/2) (5, 6) and, recently, as life-threatening organ dysfunction by a dysregulated host response to infection (sepsis-3) (7). In practice, sepsis diagnosis largely relies on clinical patient assessment (8), and biomarkers are urgently sought (9).

The recent CAPTAIN study compared the performance of 53 diverse candidate blood markers in discriminating physician-adjudicated sepsis from noninfectious SIRS on intensive care unit (ICU) admission (10). None surpassed plasma C-reactive protein (CRP). Whole blood transcriptomics revealed several multigene classifiers that may rival CRP and procalcitonin (PCT) in this clinical scenario (9, 11). They substantiated the presence of overactive innate and impaired adaptive immunity in sepsis leukocytes (12). Analysis of leukocyte subpopulations may provide further insight into the disease process. Single-cell sequencing of blood mononuclear cells, for instance, identified the early expansion of a monocytic state (MS1) in ICU patients with sepsis compared to those without sepsis. This was attributed to sepsis-induced myelopoiesis (13), a process chiefly characterized by emergency granulopoiesis (14) and leading to an increase in the ratio of nonsegmented (immature)-to-segmented (mature) neutrophils in the circulation. An increase in band cells is clinically referred to as a left shift, and further in metamyelocytes and myelocytes as a severe left shift (15). Emergency granulopoiesis is also induced in response to tissue injury by severe trauma (16). Reported changes in neutrophil phenotypes in infection and trauma in critically ill patients are thought to support both early antimicrobial activity and subsequent resolution of inflammation (17–19), including revascularization of damaged tissue (16). However, they have also been related to pathogenesis. On the one hand, different types of low-density granulocytic cells appear in the circulation that suppress T-cell responses and display other immunosuppressive features, thereby increasing the risk of nosocomial infections. One of these cell types displays a hypersegmented nucleus and low cell surface levels of CD62 (20), and another has been described as granulocytic myeloid-derived suppressor cells (MDSCs) (21). On the other hand, increased and aberrant neutrophil tissue migration, including to the lungs, liver, and kidneys, and production of reactive oxygen species, as well as support of coagulation through release of neutrophil extracellular traps, contribute to second-organ tissue damage (22, 23).

Transcriptomics studies in ICU patients admitted with and without sepsis so far have analyzed density-gradient purified blood neutrophils (24, 25). This procedure enriches mature, high-density granulocytes but depletes the abovementioned low-density immunosuppressive granulocytic populations as well as promyelocytes, myelocytes, and metamyelocytes (26). However, Nierhaus et al. (27) found that a 48% higher average count of these three early stages of terminal granulocytic differentiation together distinguished sepsis from SIRS on ICU admission (27). Transcriptional programs of early terminal granulocytic differentiation (28) may thus influence blood gene classifiers of sepsis. In a study by Parnell et al. (29), *Transcription Regulation of Granulocyte Development* was indeed the only one out of 13 immune response ontologies, overrepresented in differentially expressed genes (DEGs), that was up- rather than downregulated in whole blood RNA of ICU patients with sepsis across the first 5 days of admission compared to healthy controls (29).

Here, we aimed at identifying signature genes and pathways active in blood granulocytes, including immature precursors, that distinguish patients admitted to the ICU with sepsis and SIRS. Granulocytes were isolated based on the CD15 surface antigen that is continuously expressed throughout terminal granulocytic differentiation (30). The implications of our results for gene classifiers of sepsis are discussed.

## Methods

### Patients and Samples

Transcriptional analyses were based on two historical monocenter prospective cohorts of adults admitted to the interdisciplinary surgical ICU of a tertiary care hospital (University Medical Center Mannheim) with a recent diagnosis of SIRS or sepsis for discovery (2012–2014) (31, 32) and validation (2016–2017) (32). Samples for flow cytometric determination of CD15 blood cell counts included samples from an extended recruitment period (2018–2020) of the validation cohort. The criteria for inclusion and exclusion were detailed before (31, 32). In addition to demographic and clinical characteristics on admission (32), data on clinical phenotypes close to the time of blood sampling were retrieved from the medical records (**Supplementary Text 1**) and are summarized in **Tables 1**, **2**. Cells were selected from whole blood using CD15 MicroBeads (iltenyi Biotec, Bergisch Gladbach, Germany), preserving all stages of terminal granulocytic differentiation (32). Validation samples stored at −80°C were available for this study. Patient-level data on clinical phenotypes, characteristics of CD15^+^ cells and RNA preparations, as well as CD15 blood cell counts are available from heiDATA (https://heidata.uni-heidelberg.de/dataset.xhtml?persistentId=doi:10.11588/data/EIXOPN). Briefly, all cohorts included critically ill patients with a recent diagnosis of SIRS or sepsis according to sepsis-3 and septic shock according to sepsis-1/2. SIRS was due to surgical trauma and polytrauma, respectively, 12 and 4 times in the discovery cohort and 16 and 6 times in the validation cohort (32). In the discovery cohort, the septic focus was 11 times abdominal and 4 times pulmonary. In the validation cohort, it was 9 times each abdominal and pulmonary, twice soft tissue, and once urogenital (32). Information on the clinical phenotype of patients contributing to the flow cytometric blood count determination is included in the *Results*. The discovery cohort also included adult controls at their clinical examination a few days prior to elective surgery. The American Society of Anesthesiologists (ASA) physical status score of these presurgical controls was between 1 and 3 (mean, 1.7). One had a diagnosis of chronic obstructive pulmonary disease, four of diabetes, and two of rectal cancer.

**Table 1:** ICU patient clinical characteristics for discovery and validation of differential gene expression.

	Discovery set^a^		Validation subset A^b^			Validation subset B	
	Sepsis (*n* = 15)	SIRS (*n* = 16)	Sepsis (*n* = 18)		SIRS (*n* = 22)	Sepsis (*n* = 17)	SIRS (*n* = 15)
Demographics							
Age mean (SD) (years)	62.5 (17.5)^a^	63.1 (17.6)	69.4 (13.3)		66.3 (14.9)	72.2 (10.6)	67.9 (16.8)
Male/female patients	7/8	12/4	9/9		16/6	7/10	11/4
**Infections [*n* (%)]****^c^**							
Gram-negative bacteria	5 (33)^d^		6 (33)^e^			5 (29)	
Gram-positive bacteria	3 (20)		1 (6)			0 (0)	
Fungal	1 (7)^f^		0 (0)			0 (0)	
Viral	0 (0)		0 (0)			0 (0)	
**Treatments [*n* (%)]**							
Anti-infective^g^	13 (87)	11 (69)	14 (78)		1 (5)^****^	14 (82)	1 (7)^****^
Mechanical ventilation	15 (100)	4 (25)^****^	14 (78)		12 (55)	14 (82)	11 (73)
Renal replacement therapy	0 (0)	0 (0)	0 (0)		0 (0)	2 (12)	0 (0)
Catecholamines^h^	15 (100)	11 (69)	15 (83)		9 (41)^**^	15 (88)	8 (53)^*^
**Comorbidities [*n* (%)]****^i^**							
Renal disease	3 (20)	0 (0)	2 (11)		2 (9)	2 (12)	2 (13)
Peripheral vascular disease	1 (7)	1 (6)	8 (44)		7 (32)	7 (41)	6 (40)
Congestive heart failure	4 (27)	1 (6)	10 (56)		3 (14)^**^	10 (59)	3 (20)^*^
Any malignancy^j^	4 (27)	11 (69)^*^	8 (44)		7 (32)	6 (35)	6 (40)
Metastatic solid tumor	0 (0)	2 (13)	2 (11)		2 (9)	1 (6)	1 (7)
Plegia^k^	2 (13)	0 (0)	0 (0)		1 (5)	0 (0)	1 (7)
Diabetes with chronic complications	1 (7)	0 (0)	1 (6)		2 (9)	1 (6)	2 (13)
Diabetes without chronic complications	2 (13)	3 (19)	11 (61)		5 (23)^*^	12 (71)	5 (33)
Mild liver disease	2 (13)	0 (0)	0 (0)		2 (9)	0 (0)	2 (13)
Dementia	1 (7)	0 (0)	0 (0)		0 (0)	0 (0)	0 (0)
Chronic pulmonary disease	0 (0)	1 (6)	3 (17)		3 (14)	3 (18)	3 (20)
Myocardial infarction	0 (0)	1 (6)	0 (0)		0 (0)	0 (0)	0 (0)
Cerebrovascular disease	0 (0)	1 (6)	0 (0)		0 (0)	0 (0)	0 (0)
**SOFA score (SD)****^l^**	12.7 (3.2)	5.1 (2.4)^****^	10.8 (2.7)		6.5 (2.9)^****^	11.2 (2.3)	7.3 (2.8)^***^
**Hospital mortality [*n* (%)]**	4 (27)	0 (0) ^*^	9 (50)		5 (23)	8 (47)	5 (33)
**Blood parameters [mean (SD)]**							
CRP (mg/L)^l^	225.5 (100.9)	74.5 (47.6)^****^		312.9 (122.7)	59.1 (46.6)^****^	306.4 (114.4)	68.2 (46.1)^****^
Lactate (mmol/L)^l,m^	3.45 (2.10)	1.98 (1.91)^**^	3.01 (2.39)		1.92 (2.07)^*^	3.08 (2.44)	2.01 (2.42)^*^
Sodium (mmol/L)^n^	137.1 (4.7)	136.4 (2.8)	139.1 (5.2)		138.9 (2.7)	140.3 (5.9)	138.4 (2.8)
Total bilirubin (mg/dl)°	1.02 (0.69)	0.93 (0.53)	0.84 (0.76)		0.79 (0.92)	0.88 (0.76)	0.88 (1.11)
Creatinine (mg/dl)	1.62 (0.76)	1.20 (0.24)	1.46 (0.54)^**^		1.07 (0.60)	1.50 (0.50)^*^	1.20 (0.68)
Platelets (10^9^/L)	195.9 (110.2)	162.2 (49.6)	200.8 (108.5)		208.3 (106.5)	221.1 (97.5)	215.1 (116.1)
White blood cells (10^9^/L)	16.29 (13.03)	11.31 (3.48)	13.38 (5.26)		13.93 (4.50)	15.30 (6.33)	14.01 (4.80)

SD, standard deviation.

a Demographics of the discovery cohort were reported before (23).

b Validation subsets A and B shared 14 sepsis and 15 SIRS patients.

c Microbiology laboratory-confirmed infections.

d One patient presented combined infection with Gram-negative and Gram-positive bacteria.

e One patient presented combined infection with Gram-negative and Gram-positive bacteria.

f One patient presented a combined infection with Gram-positive bacteria and two pathogenic yeasts.

g This term indicates antibacterial, antimycotic, or antiviral drugs or combined treatment.

h This term denotes adrenalin, noradrenalin, dobutamine, or combined treatment.

i In accordance with the charted Charlson Comorbidity Index; six patients presented two or more comorbidities.

j This term includes leukemia and lymphoma.

k Hemiplegia or paraplegia.

l SOFA score, CRP, and lactate levels on admission were reported before (23).

m Lactate determinations were not available for one sepsis and three SIRS patients contributing to validation subset A and four SIRS patients contributing to both subset A and B.

n Sodium determinations were not available for one patient each contributing to validation subset A and both A and B and for one SIRS patient also contributing to both subsets.

°Total bilirubin determinations were not available for five SIRS patients from the discovery cohort, for four sepsis patients contributing to both validation subsets A and B and for one SIRS patient contributing to subset A and three to both subsets.

^****^p < 0.0001; ^***^p < 0.001; ^**^p < 0.01; and ^*^p < 0.05 after Mann–Whitney U test or Fisher’s exact test for sepsis vs. SIRS.

**Table 2:** ICU patient clinical characteristics for flow cytometric analysis of CD15 blood counts.

	Sepsis (*n* = 20)	SIRS (*n* = 14)
Demographics		
Age mean (SD) (years)	69.1 (13.2)	64.4 (12.9)
Male/female	10/10	11/3
**Infections [*n* (%)]** ^a^		
Gram-negative bacteria	4 (20)	
Gram-positive bacteria	1 (5)	
Fungal	1 (5)	
Viral	0 (0)	
**Treatments [*n* (%)]**		
Anti-infective^b^	18 (90)	0 (0)^****^
Mechanical ventilation	19 (95)	5 (36)^****^
Renal replacement therapy	2 (10)	0 (0)
Catecholamines^c^	19 (95)	5 (36)^***^
**Comorbidities [*n* (%)]** ^d^		
Renal disease	2 (10)	1 (7)
Peripheral vascular disease	4 (20)	4 (29)
Congestive heart failure	8 (40)	2 (14)
Any malignancy^e^	4 (20)	5 (36)
Metastatic solid tumor	1 (5)	0 (0)
Plegia^f^	1 (5)	0 (0)
Diabetes with chronic complications	2 (10)	0 (0)
Diabetes without chronic complications	14 (70)	3 (21)^*^
Mild liver disease	1 (5)	1 (7)
Dementia	0 (0)	0 (0)
Chronic pulmonary disease	3 (15)	1 (7)
Myocardial infarction	2 (10)	0 (0)
Cerebrovascular disease	0 (0)	0 (0)
**Hospital mortality [*n* (%)]**	8 (40)	2 (14)
**SOFA score (SD)** ^g,h^	9.8 (2.2)	6.5 (3.3)^**^
**Blood parameters [mean (SD)]**		
CRP (mg/L)^h^	242.2 (117.2)	82.4 (51.8)^****^
Lactate (mmol/L)^g,i^	2.55 (1.84)	1.72 (1.20)
Sodium (mmol/L)^j^	141.5 (4.7)	137.7 (2.6)^**^
Total bilirubin^k^ (mg/dl)	0.92 (0.67)	1.23 (1.14)
Creatinine (mg/dl)	1.55 (0.65)	1.39 (0.76)
Platelets (10^9^/L)	243.3 (141.1)	209.7 (96.2)
CRP (mg/L)^g^	242.2 (117.2)	82.4 (51.8)^****^
White blood cells (10^9^/L)^l^	14.34 (5.98)	15.55 (9.23)
**Septic focus** ^m^		
Pulmonary	10	
Abdominal	7	
Soft tissue	2	
**SIRS etiology**		
Abdominal surgery^n,o^		9
Vascular surgery^o,p^		4
Polytrauma		2

SD, standard deviation.

a Microbiology laboratory-confirmed infections.

b This term indicates antibacterial, antimycotic, or antiviral drugs or combined treatment.

c This term denotes adrenalin, noradrenalin, dobutamine, or combined treatment.

d In accordance with the charted Charlson Comorbidity Index.

e This term includes leukemia and lymphoma.

f Hemiplegia or paraplegia.

g SOFA score and CRP and lactate levels were determined on ICU admission.

h For four SIRS patients, complete data to derive the SOFA score were not available.

i For five patients with SIRS, lactate determinations were not available.

j For two SIRS patients, sodium determinations were not available.

k For two patients with sepsis and one with SIRS, determinations of total bilirubin were not available.

l For one sepsis patient, white blood cell counts were not available.

m The septic focus remained unclear in one patient.

n Includes cystectomy and esophagectomy.

°One patient had combined abdominal and vascular surgery.

p Includes aortic surgery.

^****^p < 0.0001; ^***^p < 0.001; ^**^p < 0.01; ^*^p < 0.05 after Mann–Whitney U test or Fisher’s exact test for sepsis vs. SIRS.

### Gene Expression Profiling

The discovery CD15 transcriptomes (32) was analyzed for differential gene expression by one-way analysis of variance and subjected to pathway analysis by enrichment (33) of the Kyoto Encyclopedia of Genes and Genomes (KEGG) pathways using JMP10 Genomics version 6 (SAS Institute). A false-positive rate of *α* ≤ 0.05 with false discovery rate correction (FDR-q value) was considered significant, and differential expression was assumed. A list of all DEGs and the results of the pathway enrichment analysis are available from heiDATA (https://heidata.uni-heidelberg.de/dataset.xhtml?persistentId=doi:10.11588/data/EIXOPN).

Differential expression was validated by QuantiGene Plex (QGP) assays (Thermo Fisher Scientific) (**Supplementary Table S1**). Technical duplicates were averaged. *AKIRIN1* served as a reference gene (32). Due to limited volume availability, validation samples were randomly split into overlapping subsets A and B (**Supplementary Table S1**). Expression was considered confirmed in a patient group if the assay signal was above background for at least four patients in that group. Normalized QGP results are available from heiDATA (https://heidata.uni-heidelberg.de/dataset.xhtml?persistentId=doi:10.11588/data/EIXOPN).

Differential expression of validated DEGs was assessed in two neutrophil transcriptomes available from the Gene Expression Omnibus (GEO) Profiles database (www.ncbi.nlm.nih.gov/geoprofiles) (34) under identifiers 41143967 (24) and 48169967 (25).

### Protein Localization and Function Resources

Information on the localizations and functions of proteins encoded by validated DEGs was obtained from the GeneCards database (www.genecards.org) (35). A list of 442 lysosomal genes was retrieved from The Human Lysosome Gene Database (hLGDB) (http://lysosome.unipg.it, accessed on March 20, 2019) (36). We analyzed confirmed signature genes of granule biogenesis in the microarray data for the discovery CD15^+^ cells, referring to a previous study on bone marrow maturation of human neutrophils (37). The authors used the proteome profiles of the following human neutrophil organelles, determined before by Rørvig et al. (38), as a basis:

- Primary/azurophilic granules (AG)
- Secondary/specific granules (SG)
- Gelatinase granules (GG)
- Ficolin-rich granules (FG)
- Secretory vesicles (SV)
- Cell membrane (CM).

From this, they compiled lists of signature genes encoding proteins, for which localizations to these respective compartments were supported by additional existing literature (see **Supplementary Table S6** in the online supplement to their report (37)). These lists served as a reference in our study.

### Blood Counts of Granulocyte Precursors

Blood counts of granulocyte precursors were determined in EDTA-anticoagulated blood by flow cytometry (**Supplementary Figure S1**; **Supplementary Table S2**). The results are available from heiDATA (https://heidata.uni-heidelberg.de/dataset.xhtml?persistentId=doi:10.11588/data/EIXOPN).

### Statistical Analyses

Groups and proportions were compared with the Mann–Whitney *U* test and Fisher’s exact test, respectively, using Prism 7 (GraphPad Software, San Diego). The absence of detectable gene expression by QGP in a given sample, i.e., a signal at or below background, was more frequent in the respective patient comparison group with the lower average expression level. We treated such values as missing rather than setting them to zero, i.e., background, thereby effectively reducing the sample size and following a rather conservative analytical approach. Additionally, Bonferroni adjustment was applied to QGP-validation data for DEGs with confirmed expression in both patient groups by individual selection strategy as well as to all selected DEGs with confirmed expression in both patient groups together. Differential expression was regarded as validated if statistical significance was reached for any of the strategies. In the GEO Profiles data sets, unadjusted *p*-values were calculated using *t*-tests or, in case the normality test failed, Mann–Whitney *U* tests. *p*-values <0.05 were considered significant.

Principal component analysis (PCA) was applied to the flow cytometric CD15 blood count and the QGP validation gene expression data each to obtain a condensed, lower-dimensional representation of the underlying respective data while preserving as much of the variation of the original data as possible. Here, missing values in the GPQ data were set to zero (i.e., the nominal background). PCAs were conducted using the untransformed data with centering and unit variance scaling applied to the CD15 subpopulations and QGP-validated DEGs, respectively, while singular value decomposition was employed to derive the principal components. Two-dimensional PCA plots, showing the first two principal components, are provided as an output. The analyses were performed using the programming language R (39) for statistical computing.

## Results

### Differential Gene Expression in the Discovery Set

In the discovery cohort, mean values for the sequential organ failure assessment (SOFA) score as well as blood CRP and lactate on ICU admission were significantly higher in patients with sepsis than in SIRS patients (32). Blood lactate levels on admission exceeded 2 mM for eleven out of 15 sepsis patients that thus had septic shock according to sepsis-3. Both ICU groups had higher on-admission white blood cell counts (WBCs) than presurgical patients, but higher counts in sepsis than SIRS did not reach statistical significance either on admission or close to the time of sampling. Differences in additional blood parameters between patients with sepsis and SIRS in the discovery cohort close to the time of blood sampling did not reach statistical significance. The frequencies of mechanical ventilation and hospital mortality were higher in sepsis than in SIRS. **Table 1** summarizes clinical patient characteristics of ICU patients in the discovery and validation cohorts. As an additional indicator of bacterial infection, blood PCT was routinely determined on admission in all 15 discovery cohort patients with sepsis (mean, 51.9 µg/L; range, 2.0–371.1 µg/L) but only eight out of 16 with SIRS (mean, 1.9 µg/L; range, 0.2–11.1 µg/L).

**Figure 1:**
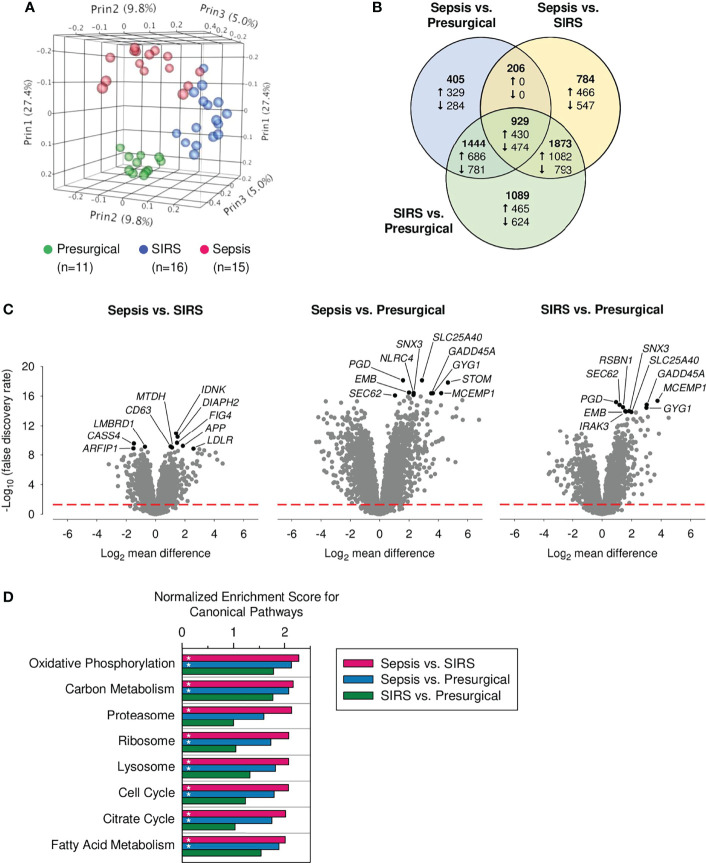
Global transcriptomic responses of discovery set CD15^+^ cells. Data from our previously published whole-genome microarray dataset (24,733 probes) for indicated numbers of presurgical, SIRS, and sepsis patients from the discovery cohort were analyzed. **(A)** Principal component analysis. Percentages represent the variance captured by the first three principal components (Prin1, Prin2, and Prin3). **(B)** Venn diagram for differentially expressed genes (DEGs) in the three pairwise patient group comparisons (total number of DEGs = 6,730; statistical significance threshold: false discovery rate adjusted *α* < 0.05). The numbers printed in bold represent the distribution of all DEGs, including up- and downregulated genes together. The numbers for the distribution of up- and downregulated genes, as per the indicated comparisons, are preceded by an upward and downward arrow, respectively. Note that, due to differences in the distributions, numbers of up- and downregulated genes add up to the numbers of all DEGs only for a given comparison, i.e., within each of the three circles. **(C)** Volcano plots for all three pairwise patient group comparisons of gene expression. The ten statistically most significant results for DEGs are identified by gene symbols. They are printed in black with the remainder in gray. The red dashed red line indicates the threshold for statistical significance. **(D)** Enrichment of canonical pathways in the discovery set CD15^+^ cells. Normalized enrichment scores for the eight CD15^+^ cell attributable KEGG pathways with the lowest false discovery rate (FDR)-q values from the sepsis vs. SIRS comparison. Bars marked with an asterisk indicate an FDR-q value <0.5.

Globally, 6,730 (27.2%) of all genes showed differential expression. In PCA analysis based on these DEGs, CD15 transcriptomes separated well into three distinct clusters (**Figure 1A**). **Figure 1B** displays relations for DEGs across the three pairwise group comparisons. Of all DEGs, 44.3% were found in the sepsis vs. presurgical, 56.3% in the sepsis vs. SIRS, and 79.3% in the SIRS vs. presurgical comparison. Shared gene expression differences in sepsis compared to SIRS and sepsis compared to presurgical controls without a change in the same direction also in SIRS compared to presurgical controls were absent (**Figure 1B**). Eight of the top ten DEGs, judged by statistical significance, were shared between the comparisons of each ICU group with the presurgical group (**Figure 1C**). However, 1.79 times more genes were differentially expressed in SIRS than in sepsis compared to presurgical controls, and only 6.0% of all DEGs were unique to the sepsis vs. presurgical comparison compared to 16.2% for SIRS vs. presurgical.

### Enrichment of Canonical Pathways in the Discovery Set

Results of the enrichment analysis for canonical pathways in the microarray data for the discovery of CD15^+^ cells revealed significant associations for similar numbers of pathways with presurgical patients compared to either ICU group, 19 for sepsis, and 21 for SIRS (**Supplementary Figure S2**). They were predominantly from the functional classes of *organismal systems* and *human diseases* and driven by high proportions of major histocompatibility complex class II genes. These pathways are not considered in more detail because they are, mainly, attributable to cell types other than granulocytes. Compared to presurgical patients, SIRS was associated with enrichment of only three and sepsis with 27 pathways, mainly *metabolism*. In a comparison of the two ICU groups, only sepsis was associated with pathway enrichment. There were in total 45 pathways, including 20 *metabolism*, 10 *genetic information processing*, and four *cellular processes*. **Figure 1D** charts normalized enrichment scores for the top eight pathways enriched in sepsis compared to SIRS. These included three pathways of mitochondrial metabolism (*oxidative phosphorylation*, *citrate cycle*, and *fatty acid metabolism*) and two of each of *genetic information processing* (*proteasome* and *ribosome*) and *cellular processes* (*lysosome* and *cell cycle*). Except for *proteasome*, these pathways were also enriched in a comparison of sepsis, but not of SIRS, to the presurgical group.

### Validation of Differential Gene Expression in Sepsis and SIRS

As with the discovery set, SOFA, CRP, and lactate values on admission were higher in sepsis than SIRS, while higher WBCs in sepsis on admission and close to the time of sampling were not statistically significant (32). In validation subsets A and B, respectively, blood lactate levels on admission were above 2 mM for eight out of 18 and nine out of 17 sepsis patients. In both subsets, serum creatinine levels were elevated in sepsis compared to SIRS in the absence of other statistically significant differences in clinical characteristics (**Table 1**). Frequencies of anti-infective and catecholamine treatments as well as congestive heart failure and diabetes without chronic complications were more frequent in patients with sepsis than in SIRS (**Table 1**). PCT on admission was available for all 21 patients with sepsis in both subsets (mean, 20.1 µg/L; range, 0.3–54.8 µg/L) but only five out of 22 with SIRS (mean, 0.8 µg/L; range, 0.3–2.0 µg/L).

To select DEGs for validation, two strategies were initially applied.

Strategy 1: The top 100 DEGs from the sepsis vs. SIRS comparison with the highest mean expression levels in sepsis and SIRS each were joined and ranked by ascending FDR-adjusted *p*-values. Forty-nine of the top 50 genes from this list (short of *MMP8*) were selected. Six additional genes were selected, for which signal ranges in the high expression group did not overlap with the 75th percentile of the low expression group and the range in the low expression group did not overlap with the 25th percentile of the high expression group.

Strategy 2: Fifty-four DEGs showed a >2-fold mean difference in sepsis and SIRS and, concomitantly, a <1.1-fold mean difference in SIRS and presurgical patients and were therefore selected.

**Figure 2:**
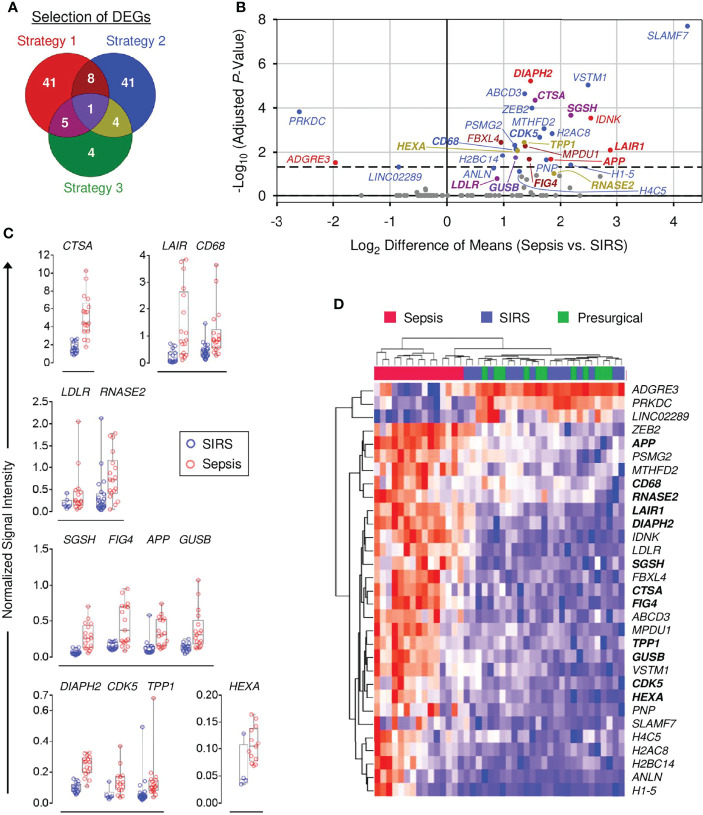
Validation of differential gene expression in sepsis and SIRS CD15^+^ cells. **(A)** Venn diagram for strategies 1–3 to select differentially expressed genes (DEGs) for validation (see main text). **(B)** QuantiGene Plex (QGP) validation results [*n* (sepsis/SIRS) = 18/22 or 17/15]. Statistical significance after Bonferroni adjustment for 93 DEGs with confirmed expression in both patient groups, out of 104 selected, is plotted vs. fold-change. Validated DEGs and their corresponding regions in the Venn diagram in **(A)** are identified by the same color code as in **(A)**. Nonvalidated DEGs are shown in gray. Names of the 13 endo-lysosomal genes are printed in bold. The dashed line indicates a globally adjusted *p*-value of 0.05. Some validated DEGs lie below this global threshold because validation was determined by the statistical test results within the separate shortlists of DEGs by strategies 1–3. **(C)** Normalized QGP signal intensity distributions for the 13 validated DEGs with endo-lysosomal associations. Group comparisons are arranged by similar intensity ranges. **(D)** Clustered heat map of validated DEGs in the discovery set microarray data (sepsis, *n* = 15; SIRS, *n* = 16; presurgical = 11). Blue indicates the minimum and red the maximum expression. The 13 endo-lysosomal genes are highlighted in bold print.

Out of the nonredundant list of 100 DEGs identified by strategies 1 and 2 (**Figure 2A**), expression in both sepsis and SIRS was confirmed for 89, and differences between these groups were validated for 30 (**Supplementary Table S3**). For 12 validated DEGs, GeneCards annotations and additional literature indicated endo-lysosomal associations: *CTSA*, *GUSB*, *HEXA*, *RNASE2*, *SGSH*, and *TPP1* encode endo-lysosomal hydrolases; *CDK5* (40) and *FIG4* (41) regulators of endo-lysosomal transport; *APP*, *CD68*, and *LAIR1* endo-lysosomal membrane proteins; and *DIAPH2* a regulator of endosome dynamics (42). This observation prompted a third selection strategy.

Strategy 3: According to hLGDB, 14 DEGs from the joined list of the top 100 DEGs with the highest mean expression levels in sepsis and SIRS were lysosomal genes. Ten were already included in strategies 1 and 2, and four were additionally selected (**Figure 2A**; **Supplementary Table S3**).

Strategy 3 led to the confirmation of expression in sepsis and SIRS for four additional genes and validation of the endo-lysosomal membrane protein-encoding *LDLR* gene, already selected by strategy 1 due to a shorter gene list and thus less conservative Bonferroni adjustment with strategy 3 than 1 (**Supplementary Table S3**). **Figure 2B** summarizes the results for the 31 validated DEGs and identifies the respective selection strategy. All but *ADGRE3*, *LINC02289*, and *PRKDC*, showed higher mean expression in sepsis than SIRS, ranging from 2.7-fold (*ANLN*) to 21.6-fold (*SLAMF7*). **Figure 2C** compares signal intensity distributions for the 13 validated DEGs with endo-lysosomal associations and **Supplementary Figure S3** for the remaining 18 validated DEGs. **Figure 2D** revisits the discovery set microarray results for validated DEGs. Only sepsis patients formed a separate cluster.

Validated DEGs were assessed for differential expression in two external microarray data sets for density-gradient purified neutrophils from patients admitted to the ICU with and without sepsis (**Supplementary Figure S4**). One featured a training and a validation cohort (24). Differences between sepsis and controls reached a statistical significance in either cohort separately and/or the pooled training and validation cohorts for a total of ten genes, including higher levels in sepsis for the endo-lysosomal genes *APP*, *CDK5*, *GUSB*, *LAIR1*, and *SGSH*. The second study compared Gram-positive, Gram-negative, and mixed sepsis to ICU controls (25). Any of the three and/or the pooled sepsis groups significantly differed from the ICU controls for a total of eleven genes, including higher sepsis levels for the endo-lysosomal genes *DIAPH2*, *GUSB*, *HEXA*, *LAIR1*, and *SGSH*.

### Key Signature Genes of Granule Biogenesis Distinguish Sepsis From SIRS

Cessation of cell proliferation and switching from oxidative to glycolytic metabolism at the metamyelocyte stage is associated with a decline in expression of cell cycle and mitochondrial genes, respectively, and additionally, proteasomal genes (43). These specific changes conspicuously match our profile of canonical pathway enrichment in sepsis (**Figure 1D**). Notably, the biogenesis of the eponymous granules during terminal granulocytic differentiation is known to depend on transcriptional waves that restrict the formation of azurophilic granules to the promyelocyte stage and of specific granules to the myelocyte/metamyelocyte stage, known as the “targeting by timing” mechanism of granule protein sorting (37, 38). Importantly, azurophilic and specific granules are lysosomal in nature. Our pathway enrichment profile (**Figure 1D**), together with validated higher expression of endo-lysosomal genes in sepsis than SIRS (**Figure 2**), led us to predict that signature genes of azurophilic and specific granule biogenesis also showed the highest expression in sepsis, reflecting elevated blood counts of immature granulocytes.

**Figure 3:**
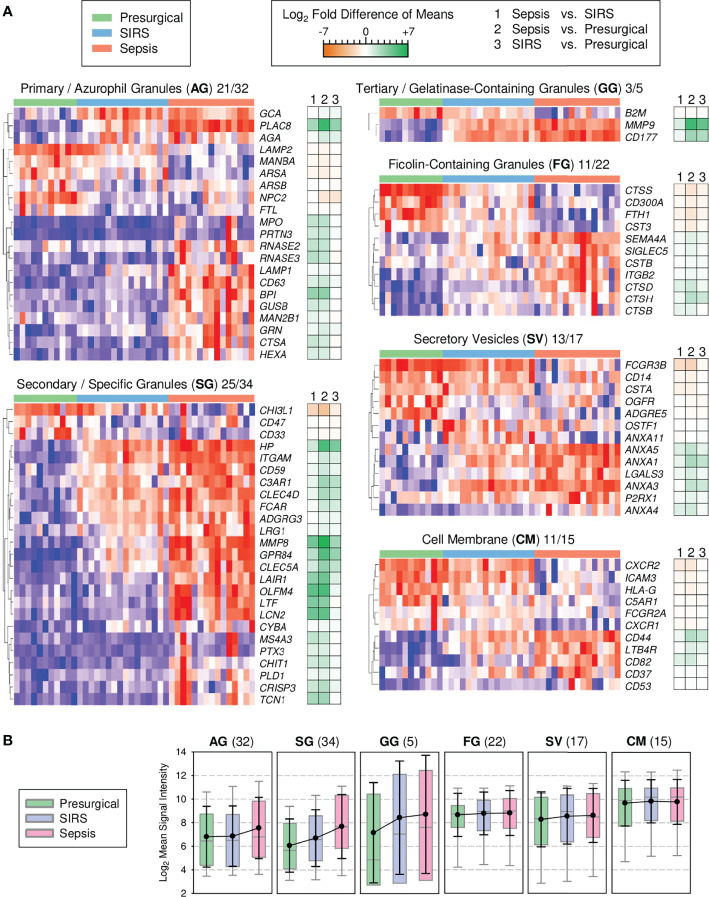
Transcriptional signatures of granule biogenesis in discovery set CD15^+^ cells (presurgical, *n* = 11; SIRS, *n* = 16, sepsis, *n* = 15). **(A)** Heat maps for granule signature genes with differential expression by subcellular compartment. Following the two-letter abbreviations for the compartments, the numbers of the shown signature genes and the full numbers of signature genes per compartment are indicated. Tiles are arranged by patient group and genes are hierarchically clustered. Blue indicates the minimum and red maximum expression. Corresponding differences of means for the three patient group comparisons are displayed as accompanying dark orange-green heat maps. **(B)** Global granule signature gene expression. Patient group means for all signature genes (number in parentheses) are summarized as colored box plots with 5th and 95th percentiles (gray whiskers) and overlaid with the global averages as black dots with standard deviations (black whiskers).

To test this, we assessed the profiles of known signature genes of granule biogenesis (37) among all 6,730 DEGs from the discovery of CD15^+^ cells (**Figure 3A**). Among the azurophilic granule genes, *PLAC8* showed, on average, the strongest fold increase in both sepsis and SIRS compared to presurgical patients. In the second main gene cluster of azurophilic granule genes, increases were more pronounced for sepsis than SIRS, with the exception of *GRN*. Among the specific granule genes, only three genes, including *CHI3L1*, showed reduced levels in both sepsis and SIRS compared to presurgical. Otherwise, profiles mostly featured gradual increases from presurgical to SIRS and sepsis patients. Three specific granule genes (*OLFM4*, *LTF*, and *LCN2*) showed <2-fold differences in SIRS vs. presurgical but >10-fold higher levels in sepsis vs. both SIRS and presurgical. The relatively small-sized gelatinase-containing granule gene profile was dominated by increases in *MMP9* and *CD177* in both sepsis and SIRS. Notably, *CD177* encodes an activation marker in chemotaxis and showed the highest increase in expression in septic compared to healthy high-density neutrophils in a previous microarray study (44). Compared to signature genes of azurophilic and specific granules as well as gelatinase-containing granules, the extent of differences between patient groups was moderate for ficolin-containing granules, secretory vesicles, and the cell membrane signature genes (**Figure 3A**).

Additionally, we assessed patient group differences by considering group averages for all individual genes within the complete set of signature genes, i.e., their global expression level, for a given compartment (**Figure 3B**). Sepsis but not SIRS was associated with a mean 1.7-fold elevation in global azurophilic granule gene expression (**Figure 3B**). Global expression of specific granule genes gradually increased by comparable margins from presurgical to SIRS and further to sepsis, while global gelatinase-containing granule gene expression was similarly elevated in both ICU groups compared to the presurgical group. There were no apparent patient group differences for the remaining compartments.

We did not formally analyze transcription factors but noted that the promyelocyte-myelocyte transition regulating *CEBPE* gene (45) showed 44% higher mean levels in sepsis than in SIRS. *CEBP* was not included in any of the selection strategies applied in this work.

### Blood Counts of Granulocyte Precursors in Sepsis and SIRS

Samples from ten patients with sepsis and four with SIRS from the validation cohort and, additionally, from ten with sepsis and ten with SIRS from an extension of the validation cohort (**Supplementary Table S4**) were subjected to flow cytometric determination of CD15 blood counts for both the precursor populations and the mature granulocytes referred to as polymorphonuclear neutrophils (PMNs) (**Supplementary Figure S1**). For the patients included in this analysis, SOFA and CRP values on admission in sepsis were higher than in SIRS, with sodium values only marginally higher. Slightly higher WBC values in SIRS than in sepsis on admission and close to the time of sampling did not reach statistical significance. Admission levels of blood lactate were above 2 mM for half of the 20 sepsis patients and one out of the 14 SIRS patients. Anti-infective treatment, mechanical ventilation, and administration of catecholamines, as well as diabetes without chronic complications were more frequent in sepsis than SIRS. The clinical characteristics of these patients are summarized in **Table 2**. Additionally, PCT on admission was available for 18 of the sepsis patients (mean, 10.5 µg/L; range, 0.3–53.8 µg/L) and 5 of the SIRS patients (mean, 1.7 µg/L; range, 0.1–3.9 µg/L).

**Figure 4:**
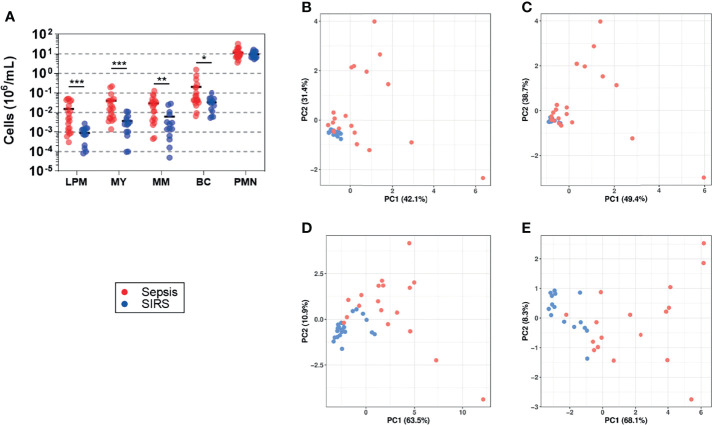
Blood counts of granulocyte precursors (sepsis, *n* = 20; SIRS, *n* = 14). **(A)** Stages of terminal granulocytic differentiation were determined in whole blood by flow cytometry (**Supplementary Figure S1**) (LPM, late promyelocyte; MY, myelocyte; MM, metamyelocyte; BC, band cell; PMN, polymorphonuclear neutrophil). Absolute counts per blood volume were determined using BD TruCount beads and are shown as scatter plots with horizontal black lines indicating group means. ^***^ *p* < 0.0005; ^**^ *p* < 0.005; ^*^ *p* < 0.05 after Mann–Whitney *U* test. PCA plots show the first two principal components (PC1 and PC2) corresponding to the CD15 cell count data shown in **(A)**, either including **(B)** or excluding **(C)** PMNs and corresponding to expression levels of genes with validated differential expression contained in validation subset A **(D)** and subset B **(E)** (**Supplementary Table S1**). The percentage of the overall variance explained by PC1 and PC2, respectively, is given in parentheses. Throughout, sepsis patients are indicated in red and SIRS patients in blue.

Throughout, blood counts of CD15 subpopulations were higher in sepsis than in SIRS (**Figure 4A**). Among the precursor populations, the mean difference was highest for late promyelocytes (16.9-fold), intermediate for myelocytes and band cells (10.9- and 11.6-fold, respectively), and lowest for metamyelocytes (6.5-fold). Only a 23% higher average count of PMNs in sepsis than in SIRS did not reach statistical significance (*p* = 0.5). PCA plots of the first two principal components suggest higher similarity among the SIRS samples in relation to the sepsis samples when based on CD15 subpopulation counts (**Figures 4B, C** **)** than on expression levels of validated DEGs (**Figures 4D, E** **)**. The two patient groups appear less similar with than without taking PMN counts into account (**Figures 4B** vs. **4C**). They also appear less similar when the analysis is based on the 13 DEGs from validation subset B compared to the 18 from subset A (**Figures 4E** vs. **4D**).

## Discussion

Whole blood transcriptomics in patients admitted to the ICU with sepsis and SIRS has resulted in the development of classifier signatures ranging from 2 to over 100 genes (9, 11, 46). Yet, the underlying differences in leukocyte subpopulation-specific responses to infection vs. sterile tissue damage remain to be dissected. In our global transcriptome analysis of CD15^+^ cells from the blood of patients with sepsis and SIRS at ICU admission and presurgical controls, these groups formed three main clusters (**Figure 1A**). Compared to presurgical controls, CD15 responses in sepsis and SIRS were overall very similar. Gene set enrichment analysis, however, revealed enrichment of a set of canonical pathways in sepsis but not in SIRS (**Figure 1D**), which is intriguingly characteristic of the promyelocyte and myelocyte stages and declines at the metamyelocyte stage (43). It includes the *cell cycle*, *oxidative phosphorylation*, *citrate cycle*, *fatty acid metabolism*, and *proteasome* pathways. The likewise enriched *ribosome* pathway may show a similar dependency on differentiation because heterochromatinization associated with loss of ribosomal protein and RNA polymerase I gene expression was described during *ex vivo* differentiation of a murine promyeloid cell line into neutrophils (47). Last but not least, our strategic selection and independent validation of genes differentially expressed in sepsis and SIRS highlighted endo-lysosomal associations in sepsis (**Figure 2**), consistent with enrichment of the *lysosome* pathway in sepsis (**Figure 1D**). In a meta-analysis of pathways in six whole blood transcriptomes, Ma et al. (48) identified *lysosome* as the top-ranking pathway associated with sepsis compared to healthy controls (48). Contrarily, the *ribosome* pathway was the top-ranking pathway associated with the controls, underscoring that the net enrichment of different gene sets (functions) in whole blood is likely determined by the contributions of different leukocyte populations.

The enrichment of canonical pathways characteristic of the promyelocyte and myelocyte stages together with validation of endo-lysosomal genes in sepsis led us to revisit known transcriptional profiles of granule biogenesis that characterize specific stages of early terminal granulocytic differentiation (37, 38) in our discovery transcriptomes (**Figure 3**). As predicted, expression of signature genes for azurophilic and specific granule biogenesis, bona fide lysosomal organelles known to be produced at the promyelocyte and myelocyte/metamyelocyte stages, respectively, increased from controls to SIRS and further to sepsis. Furthermore, azurophilic granule signature genes were elevated in sepsis but not in SIRS, among them the independently validated lysosomal genes *CTSA*, *HEXA*, *GUSB*, and *RNASE2*. This particular expression pattern is explained in the most straightforward manner by higher blood counts of both promyelocytes and myelocytes in sepsis than in SIRS, as indeed seen in an extension of the validation cohort (**Figure 4A**).

Our CD15^+^ transcriptional profiles very strongly match well-understood profiles of bone marrow promyelocytes and myelocytes (28) that both showed higher blood counts in sepsis than SIRS. From this, we conclude that genes with increased expression in sepsis compared to SIRS on ICU admission represent key signature genes of early terminal granulocytic differentiation rather than neutrophil activation as frequently concluded previously from whole blood gene classifiers of sepsis (49–51). This functional difference agrees with the above-introduced 2013 studies by Nierhaus et al. (27) and Parnell et al. (29) as well as with reported gene classifiers of sepsis that feature the azurophilic granule signature gene *PLAC8* (*FAIM3*:*PLAC8* ratio) (52) and, additionally, *LAMP1* (SeptiScore) (53).

The fact that we validated differential gene expression in CD15^+^ cells (**Figures 4B, C**; **Supplementary Figure S3**) and determined CD15 cell counts (**Figure 4A**) in samples from only partially overlapping sets of patients limits our ability to compare the discriminatory performance of these approaches in a head-to-head fashion and, thereby, our study’s direct clinical relevance. PCA analyses of our data do not yet indicate whether one approach clearly excels the other in distinguishing sepsis from SIRS (**Figures 4B–E**). Future investigation into CD15 cell-based classifiers of sepsis on ICU admission may combine CD15 subpopulation cell counts and signature gene expression. They should also resort to a quantitative PCR technique as a more sensitive and gold-standard method for gene expression analysis and be sufficiently powered. Last but not least, changes in patient characteristics over time were observed when comparing the discovery cohort (2012–2014) to the validation cohort (2016–2017) and its extension (2018–2020) (**Tables 1**, **2**). Most notably, there was an overall increase in perivascular disease, congestive heart failure, and diabetes, while the frequency of anti-infective treatment in SIRS declined, likely reflecting more restricted postoperative antibiotic prophylaxis. Changes in ICU population characteristics and clinical practice may influence the performance of sepsis classifiers.

Although the “targeting-by-timing” mechanism is well accepted for human neutrophils, we cannot exclude that timing differs between sepsis/SIRS-induced and healthy granulopoiesis for some signature genes. This may account, e.g., for the reduced rather than increased expression of the granule gene *CHI3L1* in sepsis and SIRS CD15^+^ cells. We can also not rule out that mature granulocytes contributed to the transcriptional differences. This would be consistent with the elevated expression of the validated endo-lysosomal DEGs *GUSB* and *HEXA* (azurophilic granules) as well as *LAIR1* (specific granules) in high-density blood neutrophils from ICU patients with sepsis (**Supplementary Figure S4**) (24, 25). Also, Hu et al. (54) identified the specific granule genes *CHI3L1*, *HP*, *LCN2*, and *MMP8* as hub genes in density-gradient purified blood neutrophils from patients with ARDS compared to healthy controls (54). Last but not least, other leukocyte populations may also contribute to the altered expression of granule signature genes in sepsis. The above-introduced granulocytic MDSCs also stain positive for CD15 and are characterized by the expression of matrix metalloproteinase-9 and arginase-1 (23). During terminal granulocytic differentiation, *MMP9* represents a gelatinase-containing granule signature gene (**Figure 3A**). *ARG1* is also expressed at the myelocyte/metamyelocyte stages, but arginase-1 rather localizes to azurophilic granules (38, 55). Therefore, *ARG1* is not considered a signature gene for the biogenesis of these granules. Uhel at el. ascribed elevated *MMP9* as well as *ARG1* expression in whole blood during sepsis to MDSCs (23), which may also have been present in our CD15 cell fraction. In whole blood, nongranulocytic cells may also contribute to elevated expression of granule signature genes such as *PLAC8* expressing MS1 monocytes (13).

A caveat consists of the higher SOFA scores in sepsis compared to controls on ICU admission in our (32) as well as other (56, 57) cohorts. Severe organ dysfunction in sepsis can also induce granulopoiesis and thus alter gene expression in the whole blood. Almansa et al. (58) reported a positive correlation for the expression of the granule genes *ELANE*, *MPO*, and *CTSG* (azurophilic granules) and *MMP8* (specific granules) with the SOFA score in surgical patients with sepsis (58). Moreover, Sweeney et al. (59) attributed enrichment of band cell and metamyelocyte genes in a gene classifier of acute respiratory distress syndrome (ARDS), identified in a meta-analysis of whole blood transcriptomes, to increased severity of critical illness (59). A study by Kangelaris et al. (60) that was included in that analysis specifically reported that eight genes were positively associated with ARDS in ICU patients with sepsis (60). These included *BPI* (azurophilic granules) and *HP*, *LCN2*, *MMP8*, *OLFM4*, and *TCN1* (specific granules). Bos et al. (61) also found these and, additionally, *PLAC8* (azurophilic granules) and *GPR84* and *LTF* (specific granules) upregulated in sepsis patients with a “reactive” compared to an “uninflamed” ARDS phenotype as well as enrichment of *oxidative phosphorylation* in the former (61).

In a recent notable study on whole blood gene expression in postoperative patients admitted to the ICU, cases fulfilled sepsis-3 criteria for septic shock with microbiological culture confirmation of infection, and controls met the same criteria for shock but were not infected (62). Sequential discovery by microarray and validation by RT-PCR in independent cohorts identified a six-gene classifier, including the specific granule genes *LCN2*, *LTF*, *OLFM4*, and *MMP8*, that was superior to CRP, PCT, and neutrophil counts. This supports the notion that granule signature genes can support the diagnosis of infection in patients with acute critical illness of high but comparable severity.

## Conclusions

We report an excellent match for differential CD15 cell gene and pathway expression in sepsis compared to SIRS with known promyelocyte- and myelocyte-restricted transcriptional programs. In light of elevated blood counts for these two granulocytic precursor populations in sepsis, we interpret this match to result from sepsis-induced granulopoiesis. We hence conclude that our existing process understanding of terminal granulocytic differentiation provides a rationale for the occurrence of key signature genes of this process as reported gene classifiers of sepsis in ICU patients with SIRS. Future studies of sepsis markers in blood should further assess their specificities to infection by including cases and controls with a focus on the comparability, among others, of the acuteness and degree of tissue injury and organ dysfunction.

## Data Availability Statement

The following data sets (a–d) are available from heiDATA (https://heidata.uni-heidelberg.de/dataset.xhtml?persistentId=doi:10.11588/data/EIXOPN): (a) clinical phenotype and characteristics of CD15+ cell and RNA preparations as well as flow cytometric CD15+ cell counts at the patient level, (b) list of DEGs and (c) pathway enrichment results derived from the microarray data for the discovery samples, (d) normalized QGP results for validation samples. Discovery CD15 transcriptomes are available through the GEO database (accession number GSE123731, https://www.ncbi.nlm.nih.gov/geo/query/acc.cgi?acc=GSE123731).

## Ethics Statement

The study was reviewed and approved by the Medical Ethics Commission II of the Medical Faculty Mannheim, Heidelberg University (2011-411M-MA, 2016-521N-MA). All participants or their legal representatives provided written informed consent. The discovery cohort and all patients enrolled by 2017 in the validation cohort subset A have been previously published (22, 23).

## Author Contributions

SV, TS, MT, and HL conceived the study. SV, AC, and JS performed the laboratory work. SV, AC, CS, BH, and RS contributed to the statistical analysis. SV and HL drafted the manuscript. MT and HL obtained funding. All authors participated in the acquisition, analysis, or interpretation of data and revised and approved the final version of the manuscript.

## Funding

This study was supported by the Klaus Tschira Foundation, Germany (project number 00.0277.2015). The funder had no role in study design, data collection and analysis, decision to publish, or preparation of the manuscript.

## Conflict of Interest

The authors declare that the research was conducted in the absence of any commercial or financial relationships that could be construed as a potential conflict of interest.

## Publisher’s Note

All claims expressed in this article are solely those of the authors and do not necessarily represent those of their affiliated organizations, or those of the publisher, the editors and the reviewers. Any product that may be evaluated in this article, or claim that may be made by its manufacturer, is not guaranteed or endorsed by the publisher.

## Abbreviations

ARDS, acute respiratory distress syndrome; CRP, C-reactive protein; DEG, differentially expressed gene; FDR, false discovery rate; GEO: Gene Expression Omnibus; hLGDB: Human Lysosome Gene database; ICU, intensive care unit; KEGG, Kyoto Encyclopedia of Genes and Genomes; MDSC, myeloid-derived suppressor cell; MS1, monocytic state 1; PCT, procalcitonin; QGP, QuantiGene Plex; SIRS, systemic inflammatory response syndrome; SOFA, sequential organ failure assessment; WBC, white blood cell count.
