# Supplementary material for: Key Signature Genes of Early Terminal Granulocytic Differentiation Distinguish Sepsis From Systemic Inflammatory Response Syndrome on Intensive Care Unit Admission
Source: Front Immunol. 2022 Jun 30;13:864835. doi: 10.3389/fimmu.2022.864835 (PMC9280679; doi:10.3389/fimmu.2022.864835)
Supplement: Supplementary file 1 [file DataSheet_1.pdf]

Supplementary Material

Table of Contents

Supplementary Text 1	1
Supplementary Figure 1	2
Supplementary Figure 2	3
Supplementary Figure 3	5
Supplementary Figure 4	6
Supplementary Table 1	7
Supplementary Table 2	10
Supplementary Table 3	11
Supplementary Table 4	16

SUPPLEMENTARY TEXT 1

Demographic characteristics

Our recent 2016/2017-ground truth survey conducted in the interdisciplinary surgical intensive care unit (ICU) of the University Medical Center Mannheim, the site of this study, supports the overall representativeness of our ICU patient population for Western European ICUs with regard to demographic characteristics and clinical phenotypes (Lindner et al., 2022). According to municipal population statistics (https://www.mannheim.de/de/stadt-gestalten/daten-und-fakten/bevoelkerung/einwohner-mit-migrationshintergrund, accessed on March 31 2022), the current (as of December 31, 2021) proportion of the residents of Mannheim with migrant background is at 43.6%. At least 70% of this fraction are of European ancestry. We did not record patient's racial and ethnic categories systematically. Yet, we consider our study population to be more homogenous regarding race and ethnicity than seen in hospitals that service populations with more diverse decent.

Clinical characteristics

We previously reported clinical characteristics for the discovery and validation cohorts on ICU admission (Coulibaly *et al.*, 2019). In the current study, we additionally considered available information on clinical characteristics in the medical records. The presence of infections was physician-adjudicated (T.S.). For interventions and blood marker test results, we considered the points in time nearest to the blood draws that were conducted during the routine morning laboratory orders around 7 AM. For interventions, we considered available entries recorded for the period from 7 AM on the day before the draw to 12 PM on the day of the draw. We considered entries for blood marker test results for patient samples collected between 7 AM on day 7 before the draw and 12 PM on the day of the draw.

For four of the patients with SIRS, who were enrolled during the extended recruitment period (2018–2020) of the validation cohort, and from whom samples were used in the flow cytometric analysis, blood samples were obtained in the post-anesthesia care unit, i.e., shortly prior to ICU admission. Clinical documentation for these was not available to derive the SOFA score and SAPS2 (https://heidata.uni-heidelberg.de/dataset.xhtml?persistentId=doi:10.11588/data/EIXOPN).

References

Coulibaly A, Velasquez SY, Sticht C, Figueiredo AS, Himmelhan BS, Schulte J, *et al.* AKIRIN1: A Potential New Reference Gene in Human Natural Killer Cells and Granulocytes in Sepsis. Int J Mol Sci (2019) 20(9):2290. doi: 10.3390/ijms20092290

Lindner HA, Schamoni S, Kirschning T, Worm C, Hahn B, Centner FS, *et al.* Ground Truth Labels Challenge the Validity of Sepsis Consensus Definitions in Critical Illness. J Transl Med (2022) 15;20(1): 27

SUPPLEMENTARY FIGURE 1

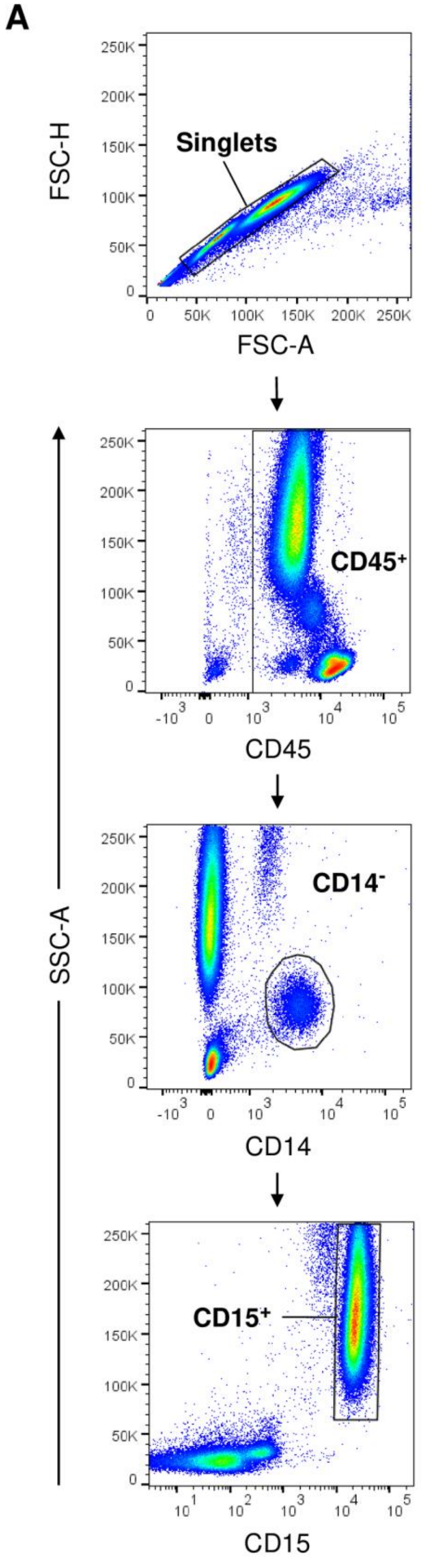

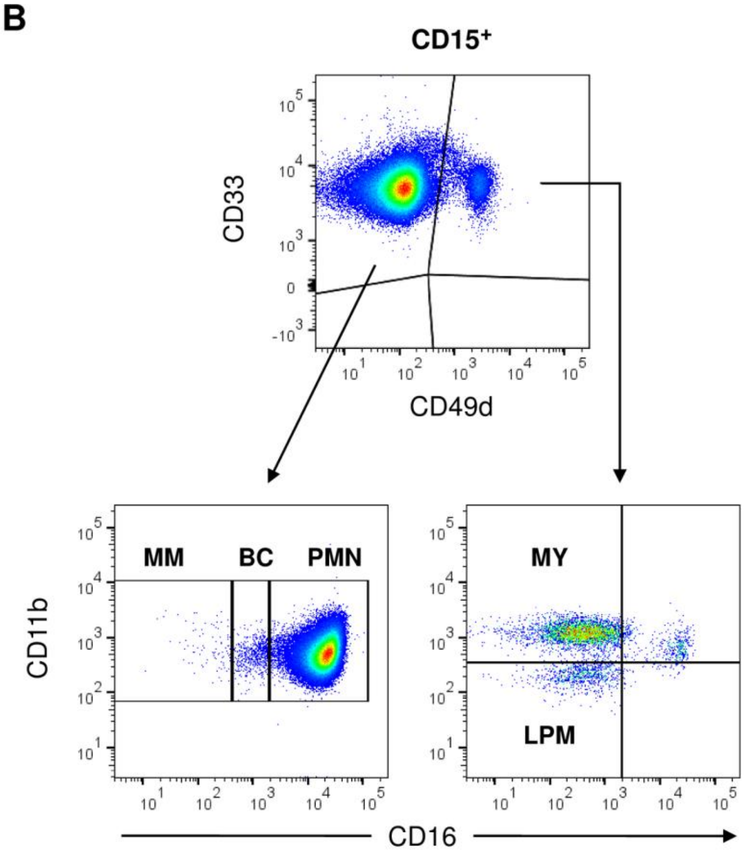

Representative dot plots illustrating the flow cytometric gating strategy for identifying stages of terminal granulocytic differentiation in whole blood (cf. Figure 5 of the main text). (**A**) Granulocytes were identified in lyzed whole blood by sequential gating on singles in a forward scatter height (FSC-H) vs. area (FSC-A) plot followed by selection of $CD45^+$ leukocytes, exclusion of $CD14^+$ monocytes and selection of $CD15^+$ granulocytes, excluding highly granular eosinophils, in sideward scatter area (SSC-A) plots. (**B**) $CD15^+$ granulocytic precursors were defined according to Pedersen et al. (J Immunol 2016; 197: 1989-1999).

**LPM**	(late promyelocyte):	$CD33^+CD49d^+CD11b^-CD16^-$
**MY**	(myelocytes):	$CD33^+CD49d^+CD11b^+CD16^-$
**MM**	(metamyelocytes):	$CD33^+CD49d^-CD11b^+CD16^-$
**BC**	(band cells):	$CD33^+CD49d^-CD11b^+CD16^{dim}$
**PMN**	(polymorphonuclear neutrophil):	$CD33^+CD49d^-CD11b^+CD16^{high}$

SUPPLEMENTARY FIGURE 2

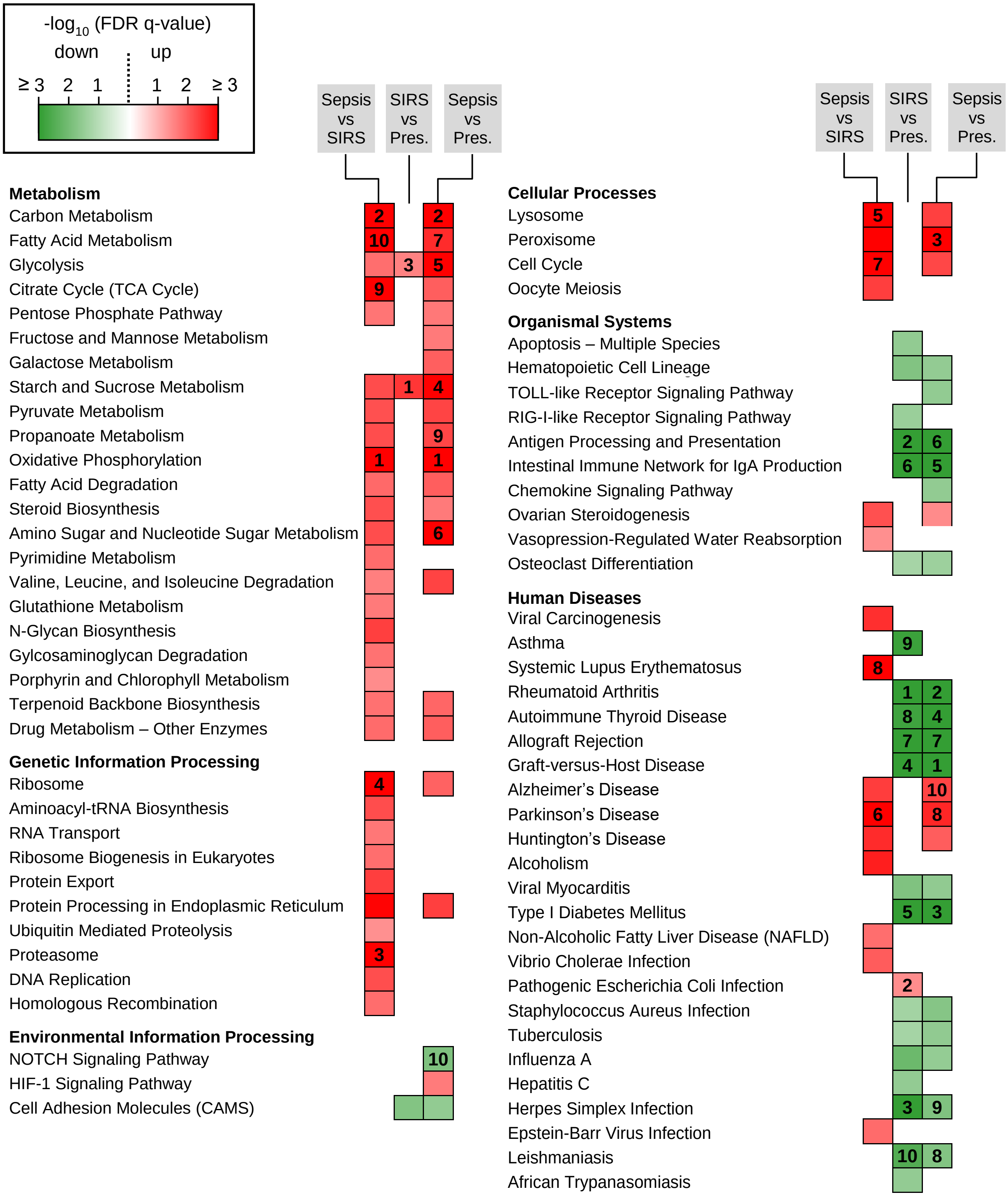

Enrichment analysis for KEGG pathways in the microarray data of discovery set $CD15^+$ cells. False discovery rate (FDR) q-values indicating statistical significance are displayed as green-red heat maps for all three pairwise patient group comparisons. Pathways are grouped by KEGG

PATHWAY database classification (https://www.genome.jp/kegg/pathway.html). For each comparison, the top ten pathways in each group, according to the normalized enrichment score (NES), are identified by the NES rank number.

SUPPLEMENTARY FIGURE 3

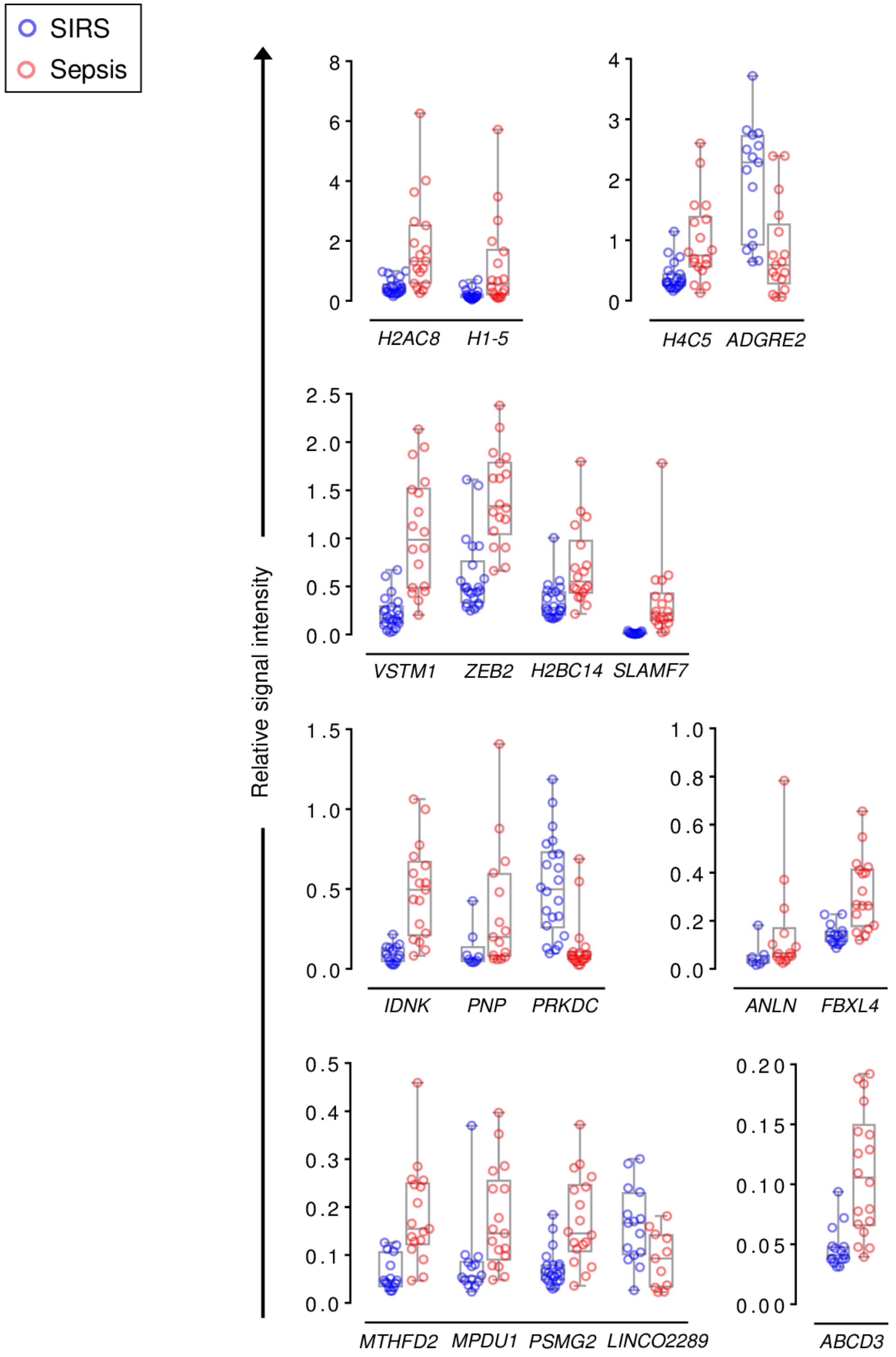

Supplemental QuantiGene Plex validation results for differential expression in sepsis and SIRS peripheral blood $CD15^+$ cells. Signal intensity distributions for the 18 validated DEGs with cellular localizations and functions other than endo-lysosomal. Group comparisons are arranged by similar intensity ranges.

SUPPLEMENTARY FIGURE 4

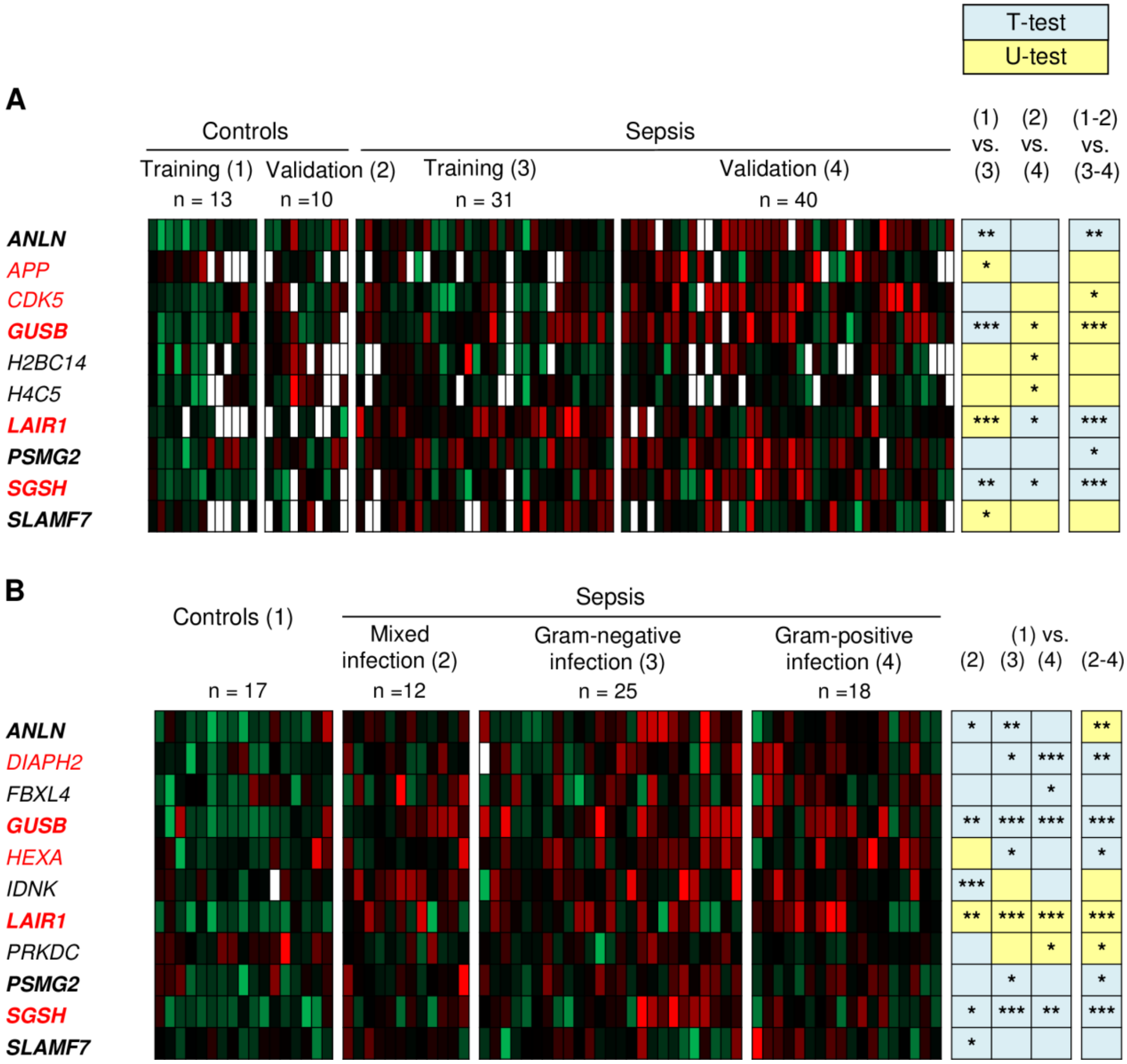

QuantiGene Plex validated genes with differential expression in density-gradient purified neutrophils from patients admitted to the ICU with and without sepsis. Curated microarray data sets were retrieved from GEO Profiles (www.ncbi.nlm.nih.gov/geoprofiles) (panel **A**: identifier 41143967; panel **B**: identifier 48169967). Tiles are arranged by patient group and genes in alphabetical order. Green indicates minimum and red maximum expression. Considered group comparisons and statistical tests used, as indicated in the main manuscript, are indicated on the right. Gene names of endo-lysosomal genes are printed in red and genes differentially expressed in both data sets (A and B) in bold. * $p<0.05$, ** $p<0.005$, *** $p<0.0005$.

SUPPLEMENTARY TABLE 1

QuantiGene Plex Assays (Thermo Fisher Scientific) identified by RefSeq IDs (https://www.ncbi.nlm.nih.gov/refseq/) and validation cohort subset with validated DEGs printed in bold.

Gene Symbol	RefSeq ID	Validation Subset
ABCD3	**NM_001122674**	**A**
ADGRE3	**NM_001289158**	**B**
AKIRIN1	NM_001136275	B
ANLN	**NM_001284301**	**A**
APP	**NM_000484**	**B**
ARFIP1	NM_001025593	B
ASPRV1	NM_152792	B
ATHL1	NM_025092	B
BCL2L15	NM_001010922	B
BPI	NM_001725	B
CASS4	NM_001164114	B
CD68	**NM_001040059**	**A**
CDCA7L	NM_001127370	B
CDK5	**NM_001164410**	**A**
CDK5R1	NM_003885	B
CEACAM1	NM_001024912	B
CEACAM6	NM_002483	B
CHIT1	NM_001256125	B
CLINT1	NM_001195555	B
CNTNAP3	NM_033655	A
CTSA	**NM_000308**	**B**
CYBB	NM_000397	B
CYP51A1	NM_000786	A
DHCR7	NM_001163817	B
DHRS9	NM_001142270	B
DIAPH2	**NM_006729**	**B**
DNM1L	NM_001278463	A
EGR1	NM_001964	A
FBN1	NM_000138	A
FBXL4	**NM_001278716**	**B**
FCHO2	NM_001146032	B
FGL2	NM_006682	B
FIG4	**NM_014845**	**B**
FLNB	NM_001164317	B
GPI	NM_000175	B
GPR137B	NM_003272	A
GPR155	NM_001033045	B

SUPPLEMENTARY TABLE 1 continued

GeneSymbol	RefSeq ID	Validation Subset
GUSB	**NM_000181**	**B**
HAL	NM_001258333	B
HCG27	NR_026791	B
HEXA	**NM_000520**	**A**
H1-5	**NM_005322**	**A**
H1-4	NM_005321	B
H2AC4	NM_003513	A
H2AC8	**NM_021052**	**A**
H2BC14	**NM_003521**	**A**
H4C5	**NM_003545**	**A**
H2BC20P	NR_036461	B
IDNK	**NM_001001551**	**B**
IKBIP	NM_153687	B
KIF27	NM_001271927	A
LAIR1	**NM_001289023**	**B**
LCN2	NM_005564	B
LDLR	**NM_000527**	**B**
LILRA1	NM_001278318	B
LOC101928143	XR_245781	B
LOC101929331	NR_121587	B
LINC02289	**NR_110553**	**B**
MPHOSPH10P1	NM_001207030	B
LOC728392	NM_001162371	B
LRRC8C	NM_032270	B
LTF	NM_001199149	B
MAK	NM_001242385	A
MARCHF8	NM_001002265	B
MICAL2	NM_001282663	B
MKI67	NM_001145966	A
MPDU1	**NM_00487**	**A**
MPO	NM_000250	B
MTHFD2	**NM_001040409**	**B**
NKG7	NM_005601	B
NLRP1	NM_001033053	B
OLFM4	NM_006418	B
PHOSPHO1	NM_001143804	B
PNP	**NM_000270**	**A**
PRAMEF9	NM_001010890	B
PRKDC	**NM_001081640**	**A**
PRTN3	NM_002777	B
PSMG2	**NM_020232**	**A**

SUPPLEMENTARY TABLE 1 continued

**GeneSymbol**	**RefSeq ID**	**Validation Subset**
REM2	NM_173527	B
RETN	NM_001193374	B
RNASE2	**NM_002934**	**A**
RPL13P5	NR_002803	B
RRM2	NM_001034	A
SCPEP1	NM_021626	B
SGMS1	NM_147156	B
SGSH	**NM_000199**	**B**
SLAMF7	**NM_001282588**	**A**
SMPDL3A	NM_001286138	B
SNORA2B	NR_002951	A
SNORD85	NR_003066	A
SNORD94	NR_004378	B
SREBF2	NM_004599	A
STOM	NM_001270526	B
SUSD3	NM_001287005	B
TARS	NM_001258437	A
TCN1	NM_001062	B
TCTEX1D1	NM_152665	B
TMCC3	NM_001301036	A
TOP2A	NM_001067	A
TPP1	**NM_000391**	**A**
TSPAN31	NM_005981	B
VSTM1	**NM_001288791**	**A**
XYLT1	NM_022166	B
ZEB2	**NM_001171653**	**A**
ZNF33A	NM_001278170	B

SUPPLEMENTARY TABLE 2

Specifications for fluorochrome-conjugated monoclonal antibodies (BD Biosciences) used in flow cytometry

Antigen	Clone	Fluorochrome
CD11b	ICRF44	FITC
CD14	MφP9	BV510
CD15	H198	APC
CD16	3G8	APC-Cy7
CD33	WM53	BV421
CD34	8G12	PE-Cy7
CD45	2D1	PerCP
CD49d	L25	PE

SUPPLEMENTARY TABLE 3

Summary of QuantiGene Plex validation results

	**Bonferroni-Adjusted *P*-Value** Selection Strategy (Validation[a] Rate)				**Median Fold Diff.**	**Protein Association[b]**			
**Gene Symbol**	1 (11/54)	2 (23/54)	3 (7/13)	1-3 (26/93)	Sepsis/ SIRS	**AG**	**SG**	**FG**	**CM**
ABCD3		<0.0001		<0.0001	2.6				
CTSA	<0.0001		<0.0001	<0.0001	2.9	X			
DIAPH2	<0.0001			<0.0001	2.8				
SLAMF7		<0.0001		<0.0001	19.0				
VSTM1		<0.0001		<0.0001	5.6				X
ZEB2		<0.0001		0.0001	2.8				
PRKDC		0.0001		0.0002	0.2				
SGSH	0.0001		<0.0001	0.0002	4.6				
IDNK	0.0002			0.0003	5.8				
MTHFD2		0.0004		0.0009	3.3				
H2AC8		0.0007		0.0015	3.6				
CDK5		0.0010		0.0022	3.1				
FBXL4	0.0021	0.0017		0.0037	1.9				
TPP1		0.0018	0.0006	0.0037	2.6			X	
PSMG2		0.0024		0.0050	2.3				
MPDU1	0.0031	0.0026		0.0054	2.6				
CD68		0.0036		0.0076	2.3				
LAIR1	0.0050			0.0085	7.4		X		
HEXA		0.0041	0.0013	0.0087	2.4	X			
H2BC14		0.0070		0.0149	2.0				
GUSB	0.0108	0.0088	0.0028	0.0187	2.3	X			
APP	0.0130			0.0225	3.6	X			
FIG4	0.0130	0.0106		0.0225	2.7				
PNP		0.0115		0.0243	3.4			X	
ADGRE3	0.0187			0.0323	0.3				
H1-5		0.0191		0.0404	4.6				
LINC02289		0.0240		0.0508	0.6				
ANLN		0.0271		0.0573	1.8				
H4C5		0.0382		0.0808	2.7				
RNASE2		0.0476	0.0152	0.1007	3.7	X			
LDLR	0.1000		0.0259	0.1722	1.8				

[a] Validation of a DEG was given if statistical significance was reached within any selection strategy.
[b] Protein associations according to Grassi *et al.* (Cell Rep 2018; 24: 2784-2794).
AG: Azurophilc Granules. SG: Secondary or Specific Granules. FG: Ficolin-Containing Granules. CM: Cell Membrane

The color scale corresponds to the p-values shown with white set to a p-value of 0.05 and increasing depth of blue and red. respectively. decreasing and increasing p-values.

SUPPLEMENTARY TABLE 3 continued

	**Bonferroni-Adjusted *P*-Value**			
	Selection Strategy			
**Gene Symbol**	1	2	3	1-3
SUSD3		0.0580		0.1225
LRRC8C		0.0615		0.1300
FLNB		0.0637		0.1346
CLINT1	0.0818			0.1409
H1-4		0.0898		0.1897
BPI	0.2535			0.4366
SMPDL3A	0.2573		0.0667	0.4431
IKBIP	0.2889			0.4976
SNORD94	0.3286			0.5660
TMCC3		0.3367		0.7116
CYBB	0.4224			0.7275
PRTN3			0.1429	0.9495
ARFIP1	1.0000			1.0000
ASPRV1	1.0000			1.0000
ATHL1	1.0000			1.0000
CASS4	1.0000			1.0000
CDCA7L		1.0000		1.0000
CDK5R1	1.0000			1.0000
CEACAM1	1.0000			1.0000
CEACAM6		1.0000		1.0000
CHIT1			0.6800	1.0000
CNTNAP3		1.0000		1.0000
CYP51A1		1.0000		1.0000
DHCR7	1.0000			1.0000
DHRS9		1.0000		1.0000
EGR1		1.0000		1.0000
FBN1		1.0000		1.0000
FCHO2[a]	1.0000			1.0000
FGL2	1.0000			1.0000
GPI	1.0000			1.0000
GPR137B		1.0000	0.8158	1.0000
GPR155	1.0000			1.0000
HAL	1.0000			1.0000
HCG27	1.0000			1.0000
H2BC20P	1.0000			1.0000
KIF27		1.0000		1.0000

SUPPLEMENTARY TABLE 3 continued

	**Bonferroni-Adjusted *P*-Value**			
	Selection Strategy			
**Gene Symbol**	1	2	3	1-3
LCN2	1.0000			1.0000
LILRA1	1.0000			1.0000
LOC101928143	1.0000	1.0000		1.0000
LOC101929331	1.0000			1.0000
MPHOSPH10P1	1.0000			1.0000
LOC728392	1.0000			1.0000
LTF	0.7637			1.0000
MAK	1.0000	1.0000		1.0000
MARCHF8	1.0000		1.0000	1.0000
MICAL2	1.0000			1.0000
MPO			0.2584	1.0000
NKG7	0.7637			1.0000
NLRP1	1.0000			1.0000
OLFM4	1.0000			1.0000
PHOSPHO1	1.0000			1.0000
REM2	1.0000			1.0000
RETN	1.0000			1.0000
RPL13P5	1.0000	1.0000		1.0000
SCPEP1			0.5112	1.0000
SGMS1	1.0000	1.0000		1.0000
STOM	1.0000			1.0000
TCN1	0.9549			1.0000
TCTEX1D1		1.0000		1.0000
TSPAN31		1.0000		1.0000
XYLT1	1.0000			1.0000
ZNF33A	1.0000	1.0000		1.0000
BCL2L15		negative		
DNM1L		negative		
H2AC4		negative		
MKI67		negative		
PRAMEF9	negative			
RRM2		negative		
SNORA2B		negative		
SNORD85		negative		
SREBF2		negative		

SUPPLEMENTARY TABLE 3 continued

	**Bonferroni-Adjusted *P*-Value**			
	Selection Strategy			
**Gene Symbol**	1	2	3	1-3
TARS		negative		
TOP2A		negative		

Negative: Signal intensities were above background for less than four patients in the sepsis and/or SIRS group.

SUPPLEMENTARY TABLE 4 ICU patient clinical characteristics for the extended recruitment period (2018–2020) of the validation cohort.

	Sepsis (n = 10)	SIRS (n = 10)
**Demographics**		
Age mean (sd) (years)	64.7 (14.5)	66.4 (12.7)
Male/Female	7/3	8/2
**Infections, n (%)**[1]		
Gram-negative bacteria	2 (20)	
Gram-positive bacteria	1 (10)	
Fungal	1 (10)	
Viral	0 (0)	
**Treatments, n (%)**		
Anti-infective[2]	9 (90)	0 (0) ***
Mechanical ventilation	10 (100)	2 (20) ***
Renal replacement therapy	0 (0)	0 (0)
Catecholamines[3]	9 (90)	3 (30) *
**Comorbidities, n (%)**[4]		
Renal disease	0 (0)	1 (10)
Peripheral vascular disease	1 (10)	3 (30)
Congestive heart failure	3 (30)	1 (10)
Any malignancy[5]	1 (10)	3 (30)
Metastatic solid tumor	0 (0)	0 (0)
Plegia[6]	1 (10)	0 (0)
Diabetes with chronic complications	1 (10)	0 (0)

SUPPLEMENTARY TABLE 4 continued

	Sepsis (n = 10)	SIRS (n = 10)
Diabetes without chronic complications	7 (70)	0 (0)**
Mild liver disease	1 (10)	0 (0)
Dementia	0 (0)	0 (0)
Chronic pulmonary disease	1 (10)	1 (10)
Myocardial infarction	2 (20)	0 (0)
Cerebrovascular disease	0 (0)	0 (0)
**Hospital mortality, n (%)**	4 (40)	1 (10)
**SOFA score (sd)**[7,8]	9.2 (2.7)	5.0 (3.0)*
**Blood parameters, mean (sd)**		
CRP (mg/L)	184.5 (78.0)	83.0 (56.9)**
Lactate (mmol/L)[8,9]	2.46 (1.20)	1.93 (1.41)
Sodium (mmol/L)[10]	142.4 (3.7)	137.6 (1.7)**
Total bilirubin (mg/dL)	1.06 (0.76)	1.02 (0.74)
Creatinine (mg/dL)	1.68 (0.82)	1.58 ± 0.82
Platelets (10^9/L)	239.9 (175.3)	196.4 (92.1)
White blood cells (10^9/L)[11]	11.79 (4.07)	16.67 (10.65)

sd = standard deviation

[1]Microbiology laboratory-confirmed infections.

[2]This term indicates antibacterial, antimycotic, or antiviral drugs or combined treatment.

[3]This term denotes adrenalin, noradrenalin, dobutamine or combined treatment.

[4]In accordance with the charted Charlson Comorbidity Index

[5]This term includes leukemia and lymphoma.

[6]Hemiplegia or paraplegia.

[7]SOFA score, CRP and lactate levels were determined on ICU admission.

[8]For four SIRS patients, complete data to derive the SOFA score were not available.

[9]For four SIRS patients, lactate determinations were not available.

[10]For two SIRS patients, sodium determinations were not available.

[11]For one sepsis patient, white blood cell counts were not available.

*** P < 0.001, ** P < 0.01, * P < 0.05 after Mann-Whitney U test or Fisher's exact test for sepsis vs. SIRS.
